# Effect of age, impaction types and operative time on inflammatory tissue reactions following lower third molar surgery

**DOI:** 10.1186/1746-160X-7-8

**Published:** 2011-04-28

**Authors:** Seidu A Bello, Wasiu L Adeyemo, Babatunde O Bamgbose, Emeka V Obi, Ademola A Adeyinka

**Affiliations:** 1 Department of Dental & Maxillofacial Surgery, State House Medical Centre, Asokoro, Abuja, Nigeria; 2 Department of Oral and Maxillofacial Surgery, Lagos University Teaching Hospital, Lagos, Nigeria; 3 College of Dentistry, University of Nebraska Medical Center, Lincoln, Nebraska, USA

## Abstract

**Background:**

Postoperative mobidity following third molar surgery is affected by a number of factors. The study of these factors is essential for effective planning and limitation of morbidity. The aim of this study was to determine the effect of age, type of impaction and operative time on immediate postoperative tissue reactions following mandibular third molar surgery.

**Methods:**

Consecutive patients with impacted mandibular third molar teeth were studied. All the third molars were classified according to Winter's classification. Surgical extraction was performed on all the patients by a single surgeon under local anaesthesia. The operation time was determined by the time lapse between incision and completion of suturing. Postoperative pain, swelling and trismus were evaluated.

**Results:**

There were 120 patients with an age range of 19-42 years. Patients in the age range of 35-42 years recorded a lower pain score (p = 0.5) on day 1. The mouth opening was much better in the lower age group on day 2 and 5 (p = 0.007 and p = 0.01 respectively). Pain, swelling and trismus increased with increasing operative time. Distoangular impaction was significantly associated with higher VAS score on day 1 and 2 (p = 0.01, 0.0, 04). Distoangular and horizontal impaction are associated with a higher degree of swelling and reduced mouth opening on postoperative review days. Vertical impaction was associated with the least degree of facial swelling and best mouth opening.

**Conclusions:**

Increasing operating time and advancing age are associated with more postoperative morbidity, likewise distoangular and horizontal impaction types.

## Background

The quality of life experienced by patients following third molar surgery is increasingly becoming a health concern [1]. Third molar surgeries are associated with unpleasant experience by the patients, referred to as postoperative morbidity, which could be divided into immediate postoperative tissue reactions and complications [2,3]. The immediate postoperative tissue reactions are characterized by pain, swelling, trismus and dysphagia [4]. Pain, swelling and trismus are normal reactions following third molar surgery and are frequent indices of researches both in the methodology of the surgery and the pharmacology of drugs used [5].

Factors affecting postoperative morbidity could be patient factors, tooth related factors and operative factors [6]. Patient factors include age, sex, size or build, ethnic background, smoking, contraceptives and oral hygiene [7]. Tooth related factors include existing infection (pericoronitis), type of impaction, depth of impaction, relationship to inferior alveolar nerve, density of surrounding bone and associated pathology like cyst or neoplasm [8]. The operative factors include the use of drugs, type and extent of incision, wound closure technique, surgeons experience and duration of operation [9,10]. Winter's classification [11] of impacted third molar is based on its orientation to an imaginary line passing through the occlusal surfaces of first and second molars to the retromolar areas as seen on a periapical radiograph (or an orthopanthomograph) [12]. The impactions are classified into horizontal, mesioangular, vertical, distoangular, buccolingual and ectopic. Pell and Gregory [13] similarly classified spatial relationship of impacted third molar into vertical, horizontal, inverted, mesioangular and distoangular.

The surrounding bone in young patients is relatively soft and more resilient compared to older patients, where the bone is harder, necessitating more bone removal, with more difficulty in separating tooth from bone, resulting in more postoperative pain, swelling and trismus [2,14]. Bruce et al, [15] while investigating the role of age on postoperative morbidity associated with mandibular third molar, found that the patients above 35 years recorded more swelling and trismus. Duration of surgery is an operative factor that has been found to influence the immediate postoperative factors following impacted third molar surgery. Definition of operating time varies among different reports. Akinwande [16] defined this as the time lapse between the beginnings of bone drilling to the end. Raprastikul et al [17] on the other hand defined it as the time lapse between incision and completion of suturing. A range of 11.03 minutes to 25.0 minutes has been reported in the literatures [16,17]. Age of patients, type of impaction and duration of operation have been mentioned in scientific literatures as factors that influence the immediate postoperative reactions following third molar surgery but objective assessment are lacking. This study aims to determine the effect of these factors on pain, swelling and trismus following mandibular third molar surgeries.

## Patients and Methods

Consecutive patients scheduled to undergo surgical removal of impacted mandibular third molars in the Maxillofacial Surgery Unit of State House Medical Centre, Abuja, Nigeria from February to November 2009, were recruited into the study. Clearance was obtained from the Ethics and Privileges Committee of the hospital and informed consent was signed by the patients before enrolment. Smokers, patient with systemic diseases and patients with active pericoronal lesions were excluded from the study. Orthopantomographic images were used to classify all the impacted mandibular third molars into Mesioangular, Distoangular, Vertical and Horizontal impactions based on Winter's classification.

Surgical extraction of a tooth per session was performed on all the patients by the same surgeon under local anaesthesia. For the patients that required bilateral extraction, a gap of at least 15 days was allowed between the two procedures to allow for total recovery from the first one. Access was gained through a 3-sided mucoperiosteal flap and ostectomy was carried out with a fissure bur and normal saline irrigation. With adequate ostectomy, elevation of the tooth was carried out and was followed by socket toileting. Sectioning of the tooth was carried out whenever necessary. They were then discharged home with standard postoperative instructions.

The operation time was determined by the time lapse between incision and completion of suturing. They were all placed on broad spectrum antibiotics and analgesics of diclofenac Potassium (cataflam Novartis) 50 mg 8 hourly for 3 days.

Postoperative pain, swelling and trismus were evaluated. Pain was estimated subjectively by asking the patient to rate the nociceptive experience on a visual analog scale of 0 to 5. The leaflets were handed over to them for daily entry with day 1 being the operation day and the assessment was done for 7 days.

**Figure 1:**
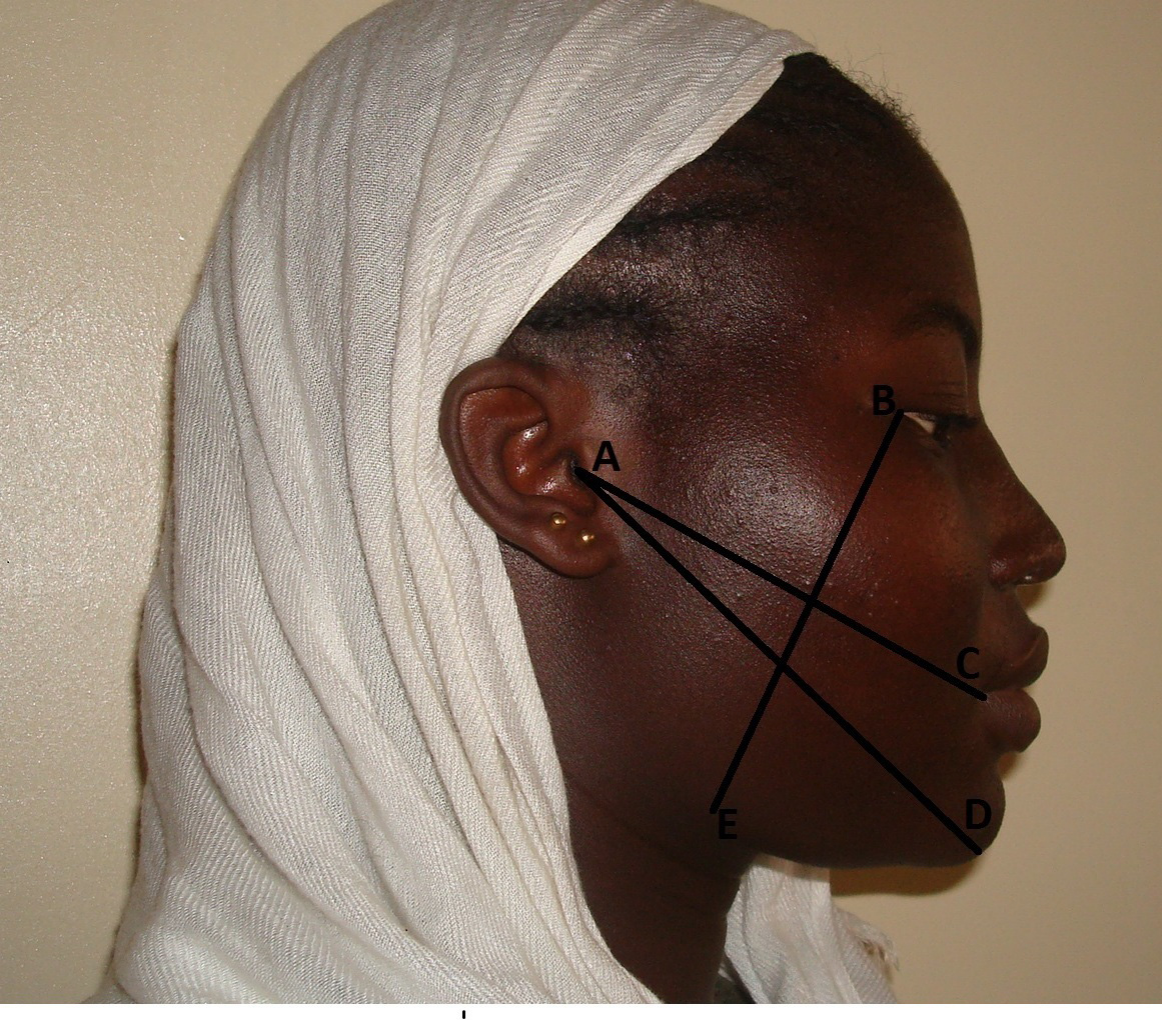
**Three-Line Facial swelling measurement**.

Swelling was assessed by a modification of a 3 line measurements (Figure 1) using 5 fixed points on surgical side of the face and finding the average. (Ustun Y, Erdogan O, Esen E, Karshi E. Comparison of the effects of 2 doses of methylprednisolone on pain, swelling and trismus after third molar surgery Oral Surg Oral Med Oral Pathol Oral Radiol Endo 2003; 96: 535-539). The fixed points used were A; the most posterior point at the midline on the tragus, B; lateral canthus of the eye, C; the most lateral point on the corner of the mouth, D; soft tissue pogonium which is the most prominent point at the midline on the chin and E; most inferior point on the angle of the mandible. The 3 lines were AC, AD and BE. A baseline measurement was carried out just before the surgery and similar measurements were carried out on days 2 (48 hours), 5 and 7 post surgery. The difference between the postoperative and preoperative measurements was calculated.

Maximum inter-incisal distance was used as the index of trismus using Boyle's gauge-a venier calibrated calliper. Percentage mouth opening was calculated on each postoperative review days. The measurement of MID was taken from the incisal edge of the upper right central incisor (or a prosthethic equivalent) to the incisal edge of the lower right central incisor. Three readings were taken for each patient and the average was determined. This constitutes the reading for the particular day. Baseline measurements were taken just before the surgery and similar readings were carried out on days 2(48 hours), 5 and 7 postoperatively. Percentage mouth opening was calculated thus: postoperative minus preoperative measurement multiplied by 100.

Data analysis was carried out with Statistical Package for Social Sciences (SPSS) 15.0 for Windows. A multivariate analysis of the effect of patients' age, sex, type of impaction and operative time on pain, swelling and trismus was also carried out. Mean values are presented with 95% confidence interval.

## Results

A total of 120 patients comprising 42 male and 78 females were studied.

**Figure 2:**
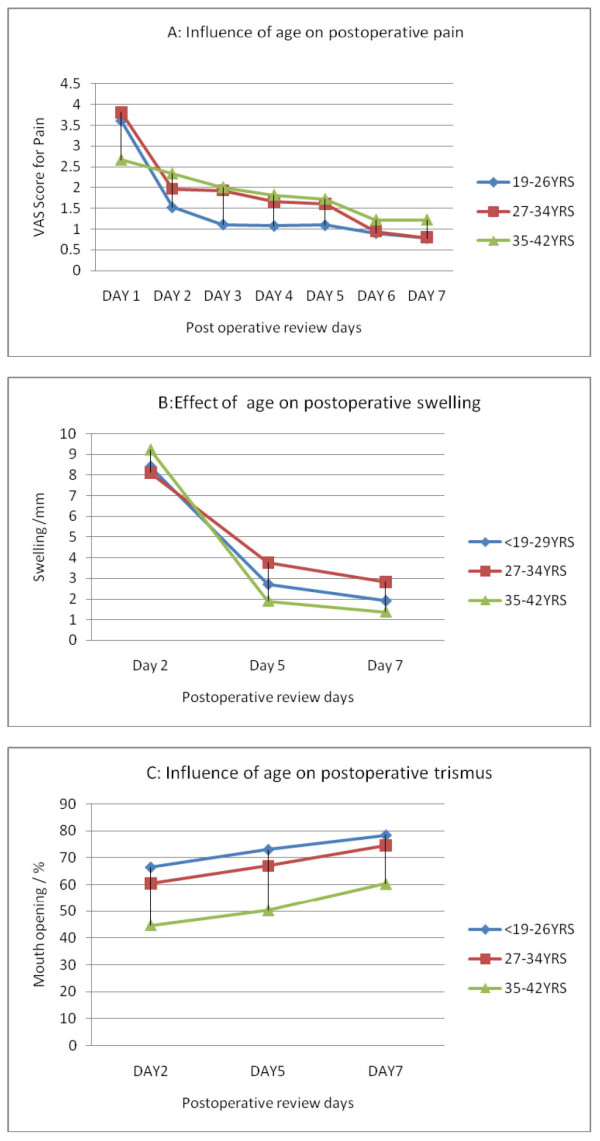
**Effect of age on postoperative pain, swelling and trismus**. A. Influence of age on Postoperative pain. B: Effect of age on Postoperative swelling. C: Influence of age on postoperative trismus.

The age range of the patients was 19 to 42 years with a mean (SD) age of 26.7 years. Fifty-eight (48.3%) patients were in the age range of 19-26 years, fifty-three (44.2%) were in the age range of 27-34 years and nine (7.5%) were in the age range of 35-42 years. The effect of age on pain, swelling and trismus is shown in Figure 2A, 2B and 2C. Patients in the age range of 35-42 years recorded a significantly lower pain score (p = 0.5) compared with lower age groups on day 1, but subsequently the pain recorded was significantly higher than that recorded for the lower age groups (P = 0.01,0.2 on day 3 and 4 respectively). The mouth opening was much better in the lower age group on day 2 and 5 (p = 0.007 and p = 0.01 respectively). There was a steady increase in the swelling recorded with increasing age but the difference in swelling between the age group ranges were not statistically significant.

The mean (SD) duration of operation was 22.63 (7.4) minutes with a range of 11 to 35 minutes. The distribution of operative time and its effect on postoperative pain, swelling and trismus is shown in tables 1 and 2. The pain increased progressively with increasing operative time on day 1 (p = 0.04). Even though the difference between the operative time ranges was not statistically significant, there was a progressive increase in swelling and trismus with increasing operative time.

**Table 1:** Influence of operative time on Postoperative pain

Operative Time/Minutes		VAS SCORE						
		DAY1	DAY2	DAY3	DAY4	DAY5	DAY6	DAY7
≤ 17	Mean	3.2667	1.6333	1.5000	1.2000	.9333	.7333	.5333
	N	30	30	30	30	30	30	30
	Std. Deviation	1.22990	1.09807	1.13715	.76112	.52083	.63968	.50742
18-23	Mean	3.3784	2.1351	1.7838	1.7568	1.6757	1.1892	.9459
	N	37	37	37	37	37	37	37
	Std. Deviation	1.55190	1.39766	1.22781	1.47959	1.56443	1.15079	1.02594
24-29	Mean	3.8421	1.2105	1.1053	.9474	1.2105	.4211	.4211
	N	19	19	19	19	19	19	19
	Std. Deviation	.83421	1.22832	.56713	.62126	.78733	.50726	.50726
30-35	Mean	4.0882	1.8235	1.3824	1.2353	1.3529	1.1471	1.1471
	N	34	34	34	34	34	34	34
	Std. Deviation	1.23993	1.40282	1.45674	1.07475	1.17763	.85749	.85749
P value		0.04	0.08	0.224	.041	.073	.005	.003

**Table 2:** Effect of operative time on postoperative swelling and trismus

Operative Time/Minutes		Swelling/mm			Mouth Opening/%		
		DAY2	DAY5	DAY7	DAY2	DAY5	DAY7
≤ 17	Mean	6.8900	2.1500	1.4567	63.6450	72.1510	79.4743
	N	30	30	30	30	30	30
	Std. Deviation	5.72968	2.65664	2.70933	18.61544	22.43444	22.35302
18-23	Mean	7.6189	3.4541	2.6649	66.5241	73.4819	81.7759
	N	37	37	37	37	37	37
	Std. Deviation	4.37168	3.73189	3.55162	23.08559	22.64894	22.45892
24-29	Mean	9.9632	4.2316	3.0158	56.5837	61.1089	67.7526
	N	19	19	19	19	19	19
	Std. Deviation	7.65014	4.31110	2.75606	15.73888	17.23444	21.27848
30-35	Mean	9.5218	2.9853	2.1441	59.2153	64.4185	68.9188
	N	34	34	34	34	34	34
	Std. Deviation	7.63341	3.61605	2.51603	20.02752	21.30764	22.86510
P value		.217	.223	.246	0.257	0.102	.032

The distribution of types of impaction and its effect on pain, swelling and trismus is shown in tables 3 and 4. Distoangular impaction is significantly associated with higher VAS score on Day 1 and 2 (p = 0.01, 0.004) when compared with the other types of impaction. Distoangular and Horizontal impaction are associated with a higher degree of swelling (p = 0.2, 0.5 and 0.0 on days 2, 5 and 7 respectively), and reduced mouth opening (p = 0.0, 0.0, 0.0 on days 2, 5 and 7 respectively) on postoperative review days when compared with vertical and mesioangular impaction. Vertical impaction was associated with the least degree of facial swelling and best mouth opening among the types of impaction

**Table 3:** Relationship of type of impaction and postoperative pain

TYPE OF IMPACTION		VASUAL ANALOGUE SCALE SCORE						
		DAY1	DAY2	DAY3	DAY4	DAY5	DAY6	DAY7
MA	Mean	3.3721	1.6744	1.4419	1.1163	.9767	.8372	.6744
	N	43	43	43	43	43	43	43
	Std. Deviation	1.34560	1.04017	.98325	.76249	.63577	.65211	.60635
V	Mean	3.2174	1.7391	1.3913	1.3913	1.2174	.8261	.7826
	N	23	23	23	23	23	23	23
	Std. Deviation	1.44463	1.32175	1.43777	1.33958	.99802	1.11405	1.12640
DA	Mean	4.3333	2.5833	1.8750	1.6667	1.6667	1.1667	1.0417
	N	24	24	24	24	24	24	24
	Std. Deviation	.81650	1.83958	1.51263	1.30773	1.09014	1.04950	.85867
H	Mean	3.7333	1.3000	1.3333	1.3667	1.6333	1.0000	.8667
	N	30	30	30	30	30	30	30
	Std. Deviation	1.33735	.91539	1.02833	1.21721	1.71169	.94686	.86037
P value		.011	.004	.372	.289	.041	.475	.380

Key

MA-Mesioangular, V-Vertical, DA-Distoangular, H-Horizontal

**Table 4:** Influence of type of impaction on postoperative swelling and Trismus

TYPE OF IMPACTION	SWELLING/mm				Mouth Opening/%		
		DAY2	DAY5	DAY7	DAY2	DAY5	DAY7
Mesioangular	Mean	7.2372	2.6814	.9070	59.4644	65.2314	71.4926
	N	43	43	43	43	43	43
	Std. Deviation	5.39210	3.21864	2juoop.04709	19.82389	22.11400	21.36244
Vertical	Mean	6.3261	1.9478	.7435	78.0052	84.9270	93.1578
	N	23	23	23	23	23	23
	Std. Deviation	6.06549	2.08324	1.54090	15.42520	15.00169	11.78812
Distoangular	Mean	8.9475	3.2417	1.6750	56.1121	63.0758	69.7213
	N	24	24	24	24	24	24
	Std. Deviation	7.23615	4.03570	2.04605	17.48335	23.35297	25.98941
Horizontal	Mean	11.0067	4.5433	4.0200	58.7123	65.4190	71.6787
	N	30	30	30	30	30	30
	Std. Deviation	6.41555	4.24408	3.50698	20.75739	19.14016	23.29330
**P value**		**.026**	**.047**	**.000**	.000	.001	.000

A multivariate analysis of the effect of patients'age, sex, type of impaction and operative time on pain, swelling and trismus is shown in table 5. Using Pillai's Trace, patients'sex, with an eigen value of 0.65, contributed least to the dependent variables of pain, swelling and trismus while operative time as a single factor, affected the dependent factors most with an eigenvalue of 1.46. Interaction of operative time and type of impaction had the highest eigenvalue of 2.66 compared to other factors matrix tests, indicating that the interactions of operative time and types of impaction affected swelling, trismus and pains observed in operated patients most. The significance of all the model matrixes (p < 0.5) is an indication that the effects of age, sex, type of impaction and operative on pain, swelling and trismus were not due to chance. However, Roy's largest root is equal to Hotelling's trace (0.352), in the case of operative time and sex interaction, which implies that sex effect does not contribute much to the output of variables of pain, swelling and trismus.

**Table 5:** Multivariate Tests of the effects of Operative time, Age range, Type of Impaction and Sex on swelling, Trismus and pain

Factor		Value	F	Hypothesis df	Error df	Sig.	Partial Eta Squared
Operative Time Range	Pillai's Trace	1.458	5.744	39.000	237.000	.000	.486
	Wilks' Lambda	.107	6.595	39.000	228.761	.000	.525
	Hotelling's Trace	3.869	7.507	39.000	227.000	.000	.563
	Roy's Largest Root	2.598	15.786(b)	13.000	79.000	.000	.722
Age Range	Pillai's Trace	.938	5.296	26.000	156.000	.000	.469
	Wilks' Lambda	.213	6.901(a)	26.000	154.000	.000	.538
	Hotelling's Trace	2.980	8.711	26.000	152.000	.000	.598
	Roy's Largest Root	2.720	16.320(b)	13.000	78.000	.000	.731
Type of Impaction	Pillai's Trace	1.075	3.395	39.000	237.000	.000	.358
	Wilks' Lambda	.211	4.054	39.000	228.761	.000	.405
	Hotelling's Trace	2.489	4.829	39.000	227.000	.000	.453
	Roy's Largest Root	1.923	11.688(b)	13.000	79.000	.000	.658
Sex	Pillai's Trace	.646	10.786(a)	13.000	77.000	.000	.646
	Wilks' Lambda	.354	10.786(a)	13.000	77.000	.000	.646
	Hotelling's Trace	1.821	10.786(a)	13.000	77.000	.000	.646
	Roy's Largest Root	1.821	10.786(a)	13.000	77.000	.000	.646
Operative Time Range * Age Range	Pillai's Trace	.879	2.520	39.000	237.000	.000	.293
	Wilks' Lambda	.308	2.863	39.000	228.761	.000	.325
	Hotelling's Trace	1.651	3.204	39.000	227.000	.000	.355
	Roy's Largest Root	1.165	7.077(b)	13.000	79.000	.000	.538
Operative Time Range * Type of Impaction	Pillai's Trace	2.655	5.008	78.000	492.000	.000	.443
	Wilks' Lambda	.009	7.520	78.000	430.658	.000	.546
	Hotelling's Trace	12.894	12.453	78.000	452.000	.000	.682
	Roy's Largest Root	9.324	58.815(b)	13.000	82.000	.000	.903
Age Range * Type of Impaction	Pillai's Trace	.591	2.518	26.000	156.000	.000	.296
	Wilks' Lambda	.490	2.539(a)	26.000	154.000	.000	.300
	Hotelling's Trace	.875	2.559	26.000	152.000	.000	.304
	Roy's Largest Root	.599	3.592(b)	13.000	78.000	.000	.374
Operative Time Range * Sex	Pillai's Trace	.261	2.087(a)	13.000	77.000	.024	.261
	Wilks' Lambda	.739	2.087(a)	13.000	77.000	.024	.261
	Hotelling's Trace	.352	2.087(a)	13.000	77.000	.024	.261
	Roy's Largest Root	.352	2.087(a)	13.000	77.000	.024	.261
Type of Impaction * Sex	Pillai's Trace	1.303	11.205	26.000	156.000	.000	.651
	Wilks' Lambda	.111	11.875(a)	26.000	154.000	.000	.667
	Hotelling's Trace	4.298	12.562	26.000	152.000	.000	.682
	Roy's Largest Root	3.090	18.540(b)	13.000	78.000	.000	.756

## Discussion

Severity of pain, amount of swelling and degree of trismus are the primary indicators of patients discomfort following surgical extraction of an impacted third molar tooth [17].

This study recorded a significant influence of age on post operative morbidity following surgical extraction of impacted third molar teeth. A higher degree of trismus and facial swelling was recorded in patients with advancing age. This finding is in agreement with some studies [15,18,19]. Bruce et al, [15] while investigating the role of age on postoperative morbidity associated with mandibular third molar, found that patients above 35 years recorded more swelling and trismus. According to de Boer [18], older patients appeared to complain of more post operative symptoms after removal of their third molar than did young patient. The reason might be that erupted molars in older patient have been used for mastication and are therefore more tightly connected to the alveolar bone by the periodontal ligament which requires more aggression to remove. Third molar surgery result in physical injury to the tissues and are therefore followed by inflammatory reaction [5,20]. It has been proposed that following tissue injury or inflammation, there is a sequential release of mediators from mast cells, the vasculature and other cells. Histamine and serotonin appear first, followed shortly after by bradykinnin and later prostaglandins and other eicosanoids. Bradykinnin has been shown to produce pain in man when given intradermally, intraarterially or intraperitoneally and the hyperalgesia associated with prostaglandin is also due to its potentiation of Bradykinnin effect [20]. Postoperative swelling results from accumulation of protein rich exudates within the surrounding tissue and trismus occurred as a result of spasm of muscle fibres following inflammatory processes. These reactions (pain, swelling and trismus) may be a consequence of the formation of prostaglandins and other mediators of inflammation derived from membrane phospholipids, which are released following surgery [5]. Chiapasco et al [2] believe that the correlation between age and post operative complications might be related to increased bone density which may result in more manipulation during the operation.

This study recorded a lower score of pain perception in the older patient than the younger ones within the first 24 hours of surgery. Pain following third molar surgery has been shown to peak within 24 hours of surgery which has equally been confirmed with this study [4]. Pain is an unpleasant sensory and emotional experience associated with actual or potential tissue damage or described in terms of such damage [21]. Information about injury or the threat of injury (due to mechanical, thermal or chemical causes) is conveyed by a specialized set of peripheral nerve fibres termed 'nociceptors', which are mainly Aδ and C fibres. Interpretation of this information (impulse) in the central nervous system is affected by many factors including earlier experience, possible concomitant stimuli from other parts of the body and individual's pain threshold [22]. This makes perception of pain very complex and subjective. Hyperalgesia which follows tissue injury and inflammation following third molar surgery, is based at least in part, on sensitization of norciceptors, but age could be a factor in which older patients have higher threshold and hence lower pain score.

The mean operation time of 22.63 recorded in this study is similar to 21.92 and 25.0 mins reported by Raprastikul et al [17] and Saglam et al [23] respectively but at variance with 11.03 mins recorded by Lopez et al [24]. Variability of operation time could be due to surgeons experience, the definition of operation time and the need for extra attachments like tube drain during the operation. A steady increase in severity of pain, trismus and swelling was observed with increased operation time despite the fact that the difference was not statistically significant. In a study on a consecutive series of 104 patients, Garcia et al [4] reported a correlation between operation time duration and analgesic use over the first 48 hours post surgery. The same finding was also reported by Perderson et al [10] while investigating the interrelation of complaints after removal of impacted mandibular third molars. The duration of operation in the hand of a single surgeon could be a reflection of the difficulty and hence duration of tissue injury associated with the operation [25]. The longer the duration of tissue injury, the more the amount of mediators released and therefore could be a reflection of the severity of pain, swelling and trismus.

The commonest type of impaction, mesioangular (n = 43,35.8%), recorded in this study is similar to the reports from earlier studies [26-28]. During normal development, the lower third molar begins its development in a horizontal angulation and as the jaw grows the angulation changes from horizontal to mesioangular then to vertical. Failure of rotation from the mesioangular to the vertical direction is the most common cause of a tooth becoming impacted [27]. Distoangular and horizontal type of impaction have been shown to be associated with higher degree of pain, swelling and trismus when compared with vertical and mesioangular type of impactions in this study. The type of impaction is an anatomical factor that determines the point of purchase (point of application of an elevator) and the extraction movements necessary to deliver a tooth during surgery [14]. The type of impaction gives a prediction of the difficulty of extraction and hence the severity of postoperative reactions. The difficulty reportedly encountered in decreasing order has been distoangular, horizontal, vertical and mesioangular [27]. Chiapasco et al [2] in their study reported 6.5% complication rate in association with distoangular impaction as opposed to 2.7% of vertical impaction. They concluded that this observation could be a reflection of surgical aggressiveness that is associated with this type of impaction.

## Conclusions

The knowledge of the effect age, operation time and type of impaction on postoperative inflammatory reactions following third molar surgery is very important because it will assist in treatment planning. It could be used as an objective tool to educate patients on the need for early extraction of an impacted third molar to minimize postoperative morbidity. The type of impaction is developmental and cannot be controlled but the knowledge could assist in objective education of patients on possible postoperative reactions for medico- legal reasons.

## Competing interests

The authors declare that they have no competing interests.

## Authors' contributions

SAB conceived of the study on third molar surgery, participated in its design and coordination and helped to draft the manuscript. EVO and AAA made substantial contributions to the design, acquisition of data and manuscript drafting. WLA and BOB participated in data analysis, interpretation and critical review of intellectual content. All authors read and approved the final manuscript.

## Authors Information

Seidu Adebayo Bello, BDS, FMCDS, FWACS,

Wasiu Lanre Adeyemo, BDS, FMCDS, FWACS, Dr. Med. Dent., FICS

Babatunde Olamide Bamgbose, BDS, FMCDS

Emeka Vitalis Obi, BDS

Ademola A Adeyinka BDS
